# AI in Musculoskeletal Imaging: An End-to-End Perspective

**DOI:** 10.3390/jcm15114077

**Published:** 2026-05-25

**Authors:** Domenico Albano, Mariachiara Basile, Stefano Fusco, Luigi Asmundo, Salvatore Gitto, Carmelo Messina, Alessio Piacentini, Francesco Rizzetto, Caterina Beatrice Monti, Moreno Zanardo, Angelo Vanzulli, Luca Maria Sconfienza

**Affiliations:** 1 Department of Radiology, ASST Grande Ospedale Metropolitano Niguarda, Piazza Ospedale Maggiore 3, 20162 Milan, Italy; luigi.asm@gmail.com (L.A.); rizzetto.fra@gmail.com (F.R.); caterinab.monti@gmail.com (C.B.M.); angelo.vanzulli@gmail.com (A.V.); 2 Dipartimento di Scienze Biomediche, Chirurgiche ed Odontoiatriche, Università Degli Studi di Milano, Via Della Commenda 10, 20122 Milan, Italy; 3 Dipartimento di Scienze Biomediche per la Salute, Università Degli Studi di Milano, Via Mangiagalli 31, 20133 Milan, Italy; mchiara1996.cb@gmail.com (M.B.); stefano.fusco23@gmail.com (S.F.); sal.gitto@gmail.com (S.G.); carmelomessina.md@gmail.com (C.M.); alessio.piacentini01@gmail.com (A.P.); io@lucasconfienza.it (L.M.S.); 4 IRCCS Ospedale Galeazzi-Sant’Ambrogio, Via Cristina Belgioioso 173, 20157 Milan, Italy; 5 U.O.C. Radiodiagnostica, ASST Centro Specialistico Ortopedico Traumatologico Gaetano Pini-CTO, P.zza Cardinal Ferrari 1, 20122 Milan, Italy; 6 Department of Life Sciences, Health and Health Professions, Link Campus University, 00165 Rome, Italy; 7 Department of Oncology and Hemato-Oncology, Università Degli Studi di Milano, Via Festa del Perdono 7, 20122 Milan, Italy

**Keywords:** artificial intelligence, musculoskeletal imaging

## Abstract

Artificial intelligence (AI) is increasingly reshaping musculoskeletal (MSK) imaging across the entire imaging pathway. This narrative review summarizes current AI applications in MSK radiology across four domains: acquisition and reconstruction, detection and triage, characterization and quantification, and prognosis and decision support. AI-based reconstruction has enabled faster MRI acquisitions, improved denoising and artifact reduction, and supported low-dose CT imaging while preserving diagnostic quality. Fracture detection and triage currently represent the most mature clinical applications, particularly in emergency settings. AI is also promoting a shift from qualitative interpretation to quantitative imaging phenotyping through automated assessment of body composition, cartilage, bone density, degenerative spine disease, skeletal maturity, and lesion heterogeneity. Emerging applications in prognostic modeling, implant evaluation, and multimodal risk stratification remain promising but less mature. Broader clinical implementation is still limited by restricted interpretability, dataset bias, insufficient prospective validation, regulatory complexity, and unresolved medico-legal issues. Overall, AI should be viewed as a tool to augment, not replace, radiological expertise.

## 1. Introduction

The use of artificial intelligence (AI) in musculoskeletal (MSK) imaging is growing rapidly. This is driven by the increasing number of orthopedic imaging studies and by the limited availability of subspecialized radiologists. AI, especially machine learning (ML) and deep learning (DL), can support pattern recognition, classification, reconstruction, and other complex tasks [1]. In radiology, its main goal is not to replace the expert, but to improve efficiency, reduce workload, and support more consistent image interpretation [2].

Although AI has shown value in several clinical areas, including trauma, oncology, and pediatric imaging, some of its most important applications in MSK radiology are found at the beginning of the imaging chain, especially in image acquisition and reconstruction [3]. DL-based methods have made it possible to accelerate MRI, particularly in knee and spine imaging, with shorter scan times and preserved image quality. In some cases, image quality may even improve [4,5,6]. At the same time, these methods also raise concerns. One important issue is that AI can generate very realistic images that may not fully reflect the patient’s true anatomy. For this reason, close collaboration between radiologists and AI developers remains essential [7].

Low-field MRI is another area in which AI may have an important role. Systems such as 0.55-T scanners are more accessible and less expensive, but image quality is lower than in standard 1.5-T or 3.0-T MRI. AI-based reconstruction can partly compensate for these limits by improving signal-to-noise ratio, spatial resolution, and contrast [8,9]. AI has also expanded the idea of image generation itself. Examples include synthetic CT from MRI for bone assessment without radiation, and synthetic fat-suppressed sequences generated from standard acquisitions [10,11]. Generative adversarial networks have further extended this field by enabling cross-modality image synthesis, which may be useful in emergency settings or when one imaging modality is not available or cannot be performed.

AI is also increasingly used for interpretative tasks, including fracture detection, lesion characterization, and tumor classification. Nevertheless, its role is not limited to image reading. AI may also support non-interpretative tasks such as workflow optimization, patient scheduling, safety, and operational efficiency [3]. It can also assist in upstream decisions, for example by helping select MRI protocols or by identifying examinations that may require intravenous contrast. This means that AI may affect not only how images are interpreted, but also how the whole MSK imaging pathway is organized. However, clinical implementation still depends on reliability, generalizability, and integration into routine practice [12,13].

This narrative review provides an overview of current AI applications in musculoskeletal imaging, with a focus on image acquisition, reconstruction, interpretation, and decision support. It also discusses the main technical, ethical, and legal challenges that still limit broader clinical adoption.

## 2. AI Along the Imaging Chain

The role of AI in MSK imaging can be understood as spanning the entire imaging pathway, from acquisition to downstream clinical decision support. To provide a structured overview, the available applications may be grouped into four broad domains: acquisition and reconstruction, detection and triage, characterization and quantification, and prognosis and decision support. Each of these areas is discussed in the following sections.

### 2.1. Acquisition and Reconstruction

AI is changing the earliest steps of the MSK imaging workflow, especially acquisition and reconstruction. The goal is to improve efficiency, standardize image quality, and reduce scan time and radiation dose. Unlike downstream tools, which mainly interpret images after they are acquired, these methods act directly on raw or incomplete data and influence how the image is generated.

One of the most important applications is MRI acceleration. DL reconstruction approaches can broadly be divided into two categories. In the post-processing one, DL is applied after images have already been reconstructed with conventional techniques. In the physics-driven one, instead, CNNs are incorporated directly into the reconstruction process and operate on raw k-space data. This second approach is particularly relevant for scan acceleration, because the AI-based reconstruction is integrated into the image-generation pipeline itself. Several MRI vendors have already patented and commercialized these technologies [14]. The accelerated protocols rely on DL reconstruction systems that use multi-scale CNNs trained on paired fully sampled and undersampled datasets to generate high-quality images. This system allows the reconstruction of missing k-space data to preserve fine anatomical details and diagnostic quality. According to several studies, DL-based reconstruction showed sensitivity and specificity of 98–100% in MSK imaging, with a slight reduction in sensitivity (91–96%) using highly accelerated protocols [4,15]. Compressed sensing combined with CNN has also reduced scan time to only a few minutes in some settings while maintaining high structural similarity and low measurement error. These developments could improve workflow efficiency in high-volume practice, boost scanner throughput, and enhance patient comfort [16].

MRI-based bone imaging is another emerging AI application in MSK imaging acquisition and reconstruction. Rather than replacing CT directly, these techniques aim to increase the amount of osseous information that can be obtained from an MRI examination while preserving its intrinsic advantage in soft-tissue assessment. Several strategies are currently available or under investigation. Ultrashort echo time and zero echo time (UTE/ZTE) sequences exploit extremely short echo times to depict tissues with very rapid signal decay, including cortical bone [17], although their spatial resolution and signal-to-noise ratio still remain inferior to CT and they do not provide native HU-based density information [18]. Other CT-like MRI techniques, such as FRACTURE, use 3D gradient-echo acquisitions to enhance bone contrast [19], but their inverted contrast may create interpretative pitfalls, particularly when cortical bone must be distinguished from air or hemorrhage [20]. A further approach is synthetic CT generation from MRI, in which DL-based image synthesis methods, including cycle-consistent generative adversarial networks (cycle GAN), can produce CT-like images from MR data [21]. This may be particularly useful in children, young patients, and longitudinal follow-up, where reducing radiation exposure is desirable. However, AI-generated bone images require careful radiological supervision, as reconstruction artifacts and hallucinated findings may lead to diagnostic errors. Therefore, MRI-based cortical bone imaging should currently be regarded as a complementary tool that may expand MRI capability in MSK imaging, rather than as a universal substitute for CT.

AI can also optimize MRI sequences. Reinforcement learning methods may adapt acquisition parameters to improve signal-to-noise ratio and tissue contrast, which is especially relevant in cartilage imaging. In addition, AI-based harmonization may reduce variability between scanners, vendors, and protocols, which is important both for clinical use and for multicenter studies [22,23].

Reconstruction has also improved through denoising and artifact reduction. DL models, including generative adversarial networks, can reduce motion-related or acquisition-related artifacts. This is relevant in MSK imaging, where long scan times and patient discomfort often affect image quality. Motion remains an important problem in other imaging modalities, including EOS imaging, and AI-based correction may help reduce clinically misleading distortions [24].

AI-based reconstruction is also increasingly used to enable low-dose imaging in CT and radiography. DL models enhance image quality from low-dose scans, reducing radiation exposure without compromising image interpretability. The implementation of DLR has the potential to reduce radiation dose by up to 45% and decrease the risk of radiation-induced cancer from 0.247% to 0.130% compared with iterative reconstruction [25,26]. Since MSK imaging often requires repeated follow-up examinations, this is particularly useful.

Finally, AI may improve image standardization by correcting variability related to patient positioning or acquisition geometry. Registration and reconstruction algorithms may reduce alignment errors and support more consistent measurements over time, which is important for longitudinal follow-up and quantitative imaging biomarkers. Ito et al. showed that AI-assisted positioning reduced workflow time (223 s vs. 255 s; *p* < 0.001) without affecting image quality or dose metrics [27].

Overall, acquisition and reconstruction are among the AI applications that are closest to routine clinical use. They work in the background, fit naturally into the imaging workflow, and can improve efficiency without removing the radiologist from the decision-making process.

### 2.2. Detection and Triage

One of the earliest and most clinically useful roles of AI in MSK imaging is detection and triage, especially in emergency and high-volume settings. Musculoskeletal trauma is one of the most common reasons for imaging in the emergency department and creates a major workload for radiologists [28]. Under these conditions, interpretation errors are not rare and may increase at night or when images are first assessed by non-radiologists. In this context, AI can help accelerate image reading, reduce missed findings, and prioritize urgent cases [29].

The most mature use case is fracture detection. AI applications in MSK trauma have involved several imaging modalities, including radiography, CT, and MRI, with radiographs representing the most extensively investigated modality to date. Progress in this field has been supported by the availability of large, anonymized image datasets, such as MURA, which includes nearly 41,000 upper-extremity radiographs classified by expert radiologists as either “normal” or “abnormal” [30].

Many studies have shown that AI can achieve diagnostic performance comparable to fellowship-trained musculoskeletal radiologists in detecting and classifying fractures, particularly in the hip, spine, and ribs [31,32,33]. Kuo et al., in their meta-analysis of 42 studies (37 on radiographs and 5 on CT), reported no statistically significant differences between clinician and AI performance in fracture detection [34]. In peripheral skeletal districts, including the knee, foot, and wrist, AI systems applied to radiographs have reported accuracies of up to 90.1%, with a measurable improvement in sensitivity among residents and less experienced readers [35]. These data support its role as a triage tool that can flag suspicious imaging studies and help prioritize reporting.

AI may be even more useful when the lesion is subtle or easy to miss. Occult fractures of the scaphoid, hip, or vertebrae may not be visible on first-line radiographs because cortical changes are minimal or technical factors limit detection [36]. In this setting, radiomics may extract texture-based features that are not visible to the human eye and may improve early detection [37,38]. This role is further supported by recent real-world data on scaphoid fractures. In a cohort including both clear-cut and more complex cases, two commercially available AI tools, BoneView and RBfracture, were evaluated. Both systems are DL tools based on DCNN using the Detectron2 framework; they process DICOM radiographs returning a categorical output (“Positive”, “Doubtful”, or “Negative”), with boxes highlighting suspected fracture sites. In definite cases, both platforms performed similarly to non-specialist radiologists (AUC~0.83 for Boneview, 0.84 for RBfracture). However, their diagnostic profiles differed: BoneView showed higher sensitivity, whereas RBfracture showed higher specificity, suggesting that different AI tools may be better suited to different clinical priorities. In more challenging cases requiring CT confirmation, performance was lower overall (AUC 0.46–0.65), but AI assistance significantly improved reader performance (AUC up to 0.75, *p* ≤ 0.03). These findings suggest that AI may be particularly useful when radiographic findings are subtle or uncertain, rather than in clearly visible fractures [39].

Recently, more technically complex DL strategies for fracture detection on radiographs were explored. For example, Tahir et al. developed an ensemble model for humeral fracture classification using the public MURA-v1.1 dataset. The model combined four ImageNet-pretrained CNN architectures, particularly VGG16, InceptionV3, MobileNetV2, and ResNet50, through a stacking approach, with Keras and TensorFlow used as foundational libraries and Google Colab as the training platform. This ensemble approach achieved 92.96% accuracy, 91.62% recall, and 92.14% F1-score, outperforming the individual modified networks. These findings suggest that combining multiple CNN architectures may improve fracture detection performance by capturing complementary image features. However, the authors also noted relevant drawbacks, including higher computational complexity, longer training time, extensive hyperparameter tuning, possible dataset bias, and limited evidence of generalizability to other anatomical regions or real-world clinical workflows [40].

Detection tools are not limited to bone trauma. On MRI, AI has also shown promise in identifying soft-tissue injuries such as anterior cruciate ligament tears and meniscal lesions. In traumatic knee imaging, early DL models such as MRNet were able to detect ACL and meniscal injuries and improved the specificity of ACL diagnosis, although performance was not uniformly superior to radiologists, with lower sensitivity for ACL tears and lower specificity for meniscal tears. More focused architectures later improved this limitation: Liu et al. used a cascaded DL-based image-cropping strategy to isolate ACL-containing slices and regions before classification, achieving sensitivity, specificity, and AUC values comparable to diagnostic radiologists. Meniscal tear detection has also been addressed with task-specific methods such as Mask-RCNN, which can support both detection and orientation classification [41]. Overall, these findings suggest that AI performance in MRI-based soft-tissue trauma depends strongly on model design, with anatomically targeted approaches likely to outperform more generic whole-image classifiers [28]. These tools may support triage and referral decisions. By contrast, more complex tasks such as predicting return to sport after ACL reconstruction are still much less mature and remain mainly investigational [42,43].

Beyond trauma, AI applications in MSK imaging also extend to the automated detection of bone lesions on CT and MRI, including metastases, myeloma-related lesions, and primary bone tumors. This may be clinically relevant in oncologic imaging, where lesions can be subtle, multifocal, and distributed across large anatomical regions, making detection time-consuming and potentially error-prone. Recent studies have mainly relied on DL models, often combined with detection or segmentation architectures such as CNNs, UNET, and YOLO. However, the current evidence remains limited [44,45,46,47].

As the number of orthopedic implant procedures continues to increase, AI-assisted postoperative image analysis may help reduce workload, limit fatigue-related errors, accelerate reporting, and improve workflow efficiency. A potential role of AI in this setting is the automatic recognition of the anatomical region, laterality, and projection, followed by detection of the orthopedic hardware. DL models have demonstrated very high accuracy in both anatomical region classification and implant detection on MSK radiographs, including knee and shoulder arthroplasties, spinal instrumentation, and fracture fixation devices, with reported accuracies approaching 100% [48,49].

Overall, AI in detection and triage should be seen as a support tool rather than a substitute for the radiologist. Among clinical AI applications in MSK imaging, this is one of the most mature areas, because it addresses a common problem, fits into daily workflow, and may reduce reporting delay and diagnostic error.

### 2.3. Characterization and Quantification

If detection is the first visible contribution of AI, its deeper impact in MSK imaging may lie in characterization and quantification. Here, AI does not simply identify an abnormality. It helps describe tissue composition, lesion heterogeneity, and disease burden in a more objective and reproducible way.

A good example is sarcopenia and body composition analysis. Traditionally, the evaluation of skeletal muscle and adipose tissue on CT has relied on manual or semi-automated segmentation [50]. This is slow, operator-dependent, and difficult to apply in routine practice. AI, especially CNN-based methods, has enabled accurate automated segmentation of skeletal muscle, subcutaneous fat, and visceral fat, most often at the L3 level. From these segmentations, clinically relevant measures can be obtained, including skeletal muscle area, skeletal muscle index, muscle attenuation, and fat distribution. DL models, especially CNN- and U-Net-based architectures, are particularly suited to image-based quantification because they can learn spatial and textural patterns directly from imaging data, reducing the need for manual feature engineering. By contrast, classical ML models, notably random forests, support vector machines, k-nearest neighbors, XGBoost, and AdaBoost, are more commonly applied to structured clinical, anthropometric, biochemical data to predict low muscle mass or sarcopenia risk, with reported AUC values around 0.81–0.90 in selected cohorts. More advanced models may also incorporate radiomic features, which can capture subtle muscle texture and composition changes that are not obvious on visual inspection. Explainable AI methods, such as SHAP, LIME, and Grad-CAM, may further improve interpretability by identifying which clinical variables or image regions contribute most to the model output. From an implementation perspective, these tools may be integrated into radiology pipelines. However, the choice of technique depends on the clinical task [51,52].

Interestingly, recent proof-of-concept data suggest that AI-based muscle assessment may not remain limited to CT. In a single-center study, a CNN trained on 1090 ultrasound images was able to classify rectus abdominis muscle density into three CT-derived categories, with an overall accuracy of 70%. Although still preliminary and limited to a single muscle and clinical setting, this approach suggests that AI may help extend body composition analysis to ultrasound, potentially making muscle quality assessment more accessible, portable, and suitable for bedside evaluation [53].

Degenerative disease is a clear example of this shift toward reproducible quantification. In osteoarthritis, conventional radiographic grading systems such as Kellgren-Lawrence remain widely used, but they are semi-quantitative, subjective, and relatively insensitive to early disease. DL models can automate radiographic grading and improve reproducibility by learning image patterns related to joint space narrowing, osteophytes, bone shape, and subchondral changes. In a representative study, Tiulpin et al. developed a transparent computer-aided diagnosis model for knee osteoarthritis grading on plain radiographs using a Deep Siamese CNN trained on the Multicenter Osteoarthritis Study dataset and externally validated on the Osteoarthritis Initiative dataset. The model was implemented in PyTorch and trained on GPU hardware, using an ensemble strategy and Grad-CAM-based attention maps to make the decision process more interpretable. Compared with a generic transfer-learning baseline based on fine-tuned ResNet-34, the Siamese architecture achieved similar overall performance but produced better classification performance of early osteoarthritis (AUC 0.93), suggesting that anatomically constrained architectures may be preferable to generic image-classification networks in fine-grained radiographic grading tasks [54].

Beyond radiographic analysis for the automatic detection of osteoarthritis severity, DL models may also improve the MRI-based automated cartilage segmentation for quantitative T2 mapping. When integrated into standard MRI protocols for morphological assessment, this approach can provide information on the morphology and local biochemical composition of cartilage, supporting osteoarthritis assessment and helping early identification of individuals at risk of disease progression, when preventive measures may still be implemented [55].

On MRI, AI can go further by segmenting cartilage, menisci, and bone marrow lesions and by quantifying cartilage thickness, volume, and compositional biomarkers. Models such as U-Net and 3D convolutional networks have shown high segmentation performance. AI can also speed up semiquantitative scoring systems such as WORMS and MOAKS, reducing reading time while maintaining good agreement with expert readers [56].

A similar change is also taking place in spine imaging. Degenerative spine disease is very common, and its interpretation often presents some variability among different readers. DL models have been developed to allow automatic labeling of lumbar levels and detection of findings such as disc degeneration, spinal canal stenosis, spondylolisthesis, disc bulging, and nerve root compression. MRI-based markers, such as the Vertebral Bone Quality (VBQ) score and the M-score, also allow for opportunistic assessment of bone quality and osteoporosis by quantifying bone marrow fat changes on T1-weighted images. Although agreement with experts can be good, performance may decrease in anatomically complex regions such as L4–L5, reinforcing the need for radiologist oversight [57,58,59]. This quantitative approach can also be extended to CT. Both ML- and DL-based methods applied to CT imaging have demonstrated increasing potential to provide automated, reproducible, and scalable assessment of bone-related parameters. In a landmark study including more than 500,000 CT examinations from over 280,000 patients, DL models enabled fully automated vertebral segmentation, three-dimensional ROI placement, and quantification of trabecular attenuation across multiple scanners and acquisition protocols, with excellent agreement with expert radiologists (>99%) [60]. Moreover, in a recent study evaluating seven ML models using L1–L4 average HU value as the sole input variable, the K-nearest neighbors model showed balanced and robust performance (accuracy: 0.71), whereas Logistic Regression and Naive Bayes achieved high AUCs (0.78), supporting the use of CT-derived HU values as a simple and practical tool for opportunistic osteoporosis screening, particularly when DXA is unavailable or limited [61].

AI may also be used to automate spinal measurements on radiographs. In a recent multi-reader study, an AI algorithm measured key spinopelvic parameters, including thoracic kyphosis, lumbar lordosis, sacral slope, and sagittal vertical axis, with accuracy comparable to experienced radiologists and surgeons, (ICC 0.92–1.00; no significant differences vs. readers, *p* > 0.05), while markedly reducing reading time. Notably, its performance remained strong even in the presence of vertebral fractures or spinal instrumentation, suggesting that AI could improve reproducibility in routine spinal assessment [62].

Radiomics plays an important role in MSK oncology. By extracting quantitative features related to texture, shape, intensity, and spatial heterogeneity, radiomics may help distinguish aggressive from non-aggressive lesions, improve tumor grading, and better characterize cartilaginous, lipomatous, and sarcomatous lesions. From a methodological perspective, most AI studies in MSK oncology still rely on radiomics-based ML models applied to CT and MRI, whereas DL represents a smaller but growing proportion of the literature. Radiomics requires predefined steps, including image acquisition, manual or semi-automated segmentation, feature extraction, feature selection, and classifier development. Texture analysis may reveal intratumoral heterogeneity that is not visible to the human eye but may reflect biological aggressiveness. In osteosarcoma, for example, habitat imaging, histogram analysis, and delta-radiomics may describe different tumor subregions and monitor treatment-related changes [63]. In this workflow, ML is used to perform classification tasks. In MSK oncology, conventional ML, particularly radiomics-based ML applied to CT and MRI, has been largely explored for benign versus malignant discrimination, grading of cartilaginous tumors and soft-tissue sarcomas, and prediction of therapy response, recurrence, and survival. In contrast, DL may outperform conventional ML in classification tasks. DL models can learn image features directly from radiographs, CT, or MRI, reducing the need for handcrafted feature engineering, but they generally require larger datasets and greater computational resources compared to conventional ML. This explains why their application remains limited in rare MSK tumors. However, DL models have been applied to primary bone tumor classification on radiographs, including benign versus malignant lesions discrimination or grading, showing better accuracy compared to radiology residents [64]. DL was also applied for automated osteosarcoma segmentation in CT images [65]. Finally, radiomics-based DL models have been used for both classification and segmentation tasks, to distinguish lung from non-lung spine bone metastases on dynamic contrast-enhanced MRI or benign from malignant sacral tumors on CT.

Overall, current evidence remains limited by small sample sizes, manual segmentation variability, tumor rarity, and the lack of external validation. Public repositories such as The Cancer Imaging Archive (https://www.cancerimagingarchive.net, accessed on 20 May 2026) and multicenter institutional infrastructures may help overcome these limitations by providing access to independent datasets for model training and validation. In tumor characterization and quantification, AI should be interpreted not as a single technique but as a spectrum ranging from handcrafted radiomics and conventional ML to DL-based automated feature learning, each with different advantages, data requirements, and barriers to clinical implementation [66,67,68,69].

Bone age assessment also belongs in this section. It is one of the most established and clinically relevant applications of AI in musculoskeletal imaging, with implications spanning pediatric endocrinology, orthopedics, and even legal medicine. Traditionally, skeletal maturity is evaluated using atlas-based or scoring systems, most notably the Greulich-Pyle (GP) and Tanner-Whitehouse (TW) methods [70,71], both relying on morphological analysis of epiphyseal and diaphyseal development on hand and wrist radiographs. Despite their widespread use, these approaches are inherently time-consuming and subject to significant intra- and inter-observer variability. In this context, bone age estimation has emerged as a prototypical task for AI, as it can be framed as a combination of object detection, segmentation, and classification problems. Recent advances in ML and DL have enabled the development of highly accurate and fully automated solutions [72].

Among these, BoneXpert represents one of the most widely adopted systems in clinical practice [73]. It performs automated segmentation and analysis of multiple bones extracting morphological and textural features to compute skeletal maturity, which can then be converted into GP or TW-equivalent bone age values. Similarly, newer DL-based platforms, such as VUNO Med-BoneAge [74], employ CNN trained on large datasets of pediatric radiographs to provide rapid and reproducible estimates of bone age. These systems can deliver results within seconds and demonstrate high concordance with expert radiologist assessments, with correlation coefficients typically exceeding 0.8 and mean differences on the order of a few months [75,76].

A key advantage of AI-based bone age assessment lies in its ability to reduce observer dependency and improve workflow efficiency. Studies have consistently shown that automated systems not only decrease reporting time but also significantly reduce intra-reader variability, yielding more standardized and reproducible measurements across different levels of expertise. These systems reduce reader variability and save time, although performance may still vary across different populations and in patients with abnormal skeletal development [77].

AI may also be applied to orthopedic implant characterization and quantitative assessment. DL models, particularly CNN, have shown high performance in detecting the presence of arthroplasty on radiographs and in classifying implant design types, such as total versus unicompartmental knee arthroplasty or anatomical versus reverse total shoulder arthroplasty. Similar models have also been used to identify specific implant models, a task that is especially relevant before revision surgery, where correct implant recognition may guide the selection of dedicated surgical instruments and reduce reliance on time-consuming implant atlases. Indeed, ResNet-based DCNNs implemented in PyTorch achieved excellent performance for knee and shoulder implant classification, with class activation maps confirming that the models focused on relevant prosthetic components rather than unrelated image features [78,79].

Beyond classification, AI can support quantitative postoperative assessment. U-Net-based segmentation models, developed in Python/TensorFlow and combined with image-processing libraries, have been used to automatically measure acetabular inclination and anteversion after total hip arthroplasty. This approach achieved measurements close to human readers. From a practical perspective, classification models appear computationally faster and well suited for implant detection or model recognition, whereas segmentation-based pipelines are more demanding but provide clinically useful quantitative outputs [80,81]. AI has also been explored for implant-related complications, particularly prosthetic loosening. CNN models were trained to detect loosening from preoperative radiographs of patients undergoing revision total hip or knee arthroplasty, using intraoperative findings as the reference standard. A model with only the radiological image as input achieved 70% accuracy, while a final DenseNet-based model combining anteroposterior and lateral radiographs with clinical history reached 88.3% accuracy. These results suggest that AI may detect prosthetic loosening from radiographs, especially when imaging and clinical data are integrated [82].

Overall, AI in orthopedic implant evaluation is moving from simple recognition toward automated, reproducible characterization and measurement, although broader validation across implant types, imaging protocols, and institutions remains necessary before routine clinical adoption.

Taken together, these applications show that one of the most important effects of AI in MSK imaging is the shift from qualitative reading to quantitative phenotyping. This area is highly promising, but its clinical value still depends on robust segmentation, good external validation, and consistent performance across scanners and populations.

### 2.4. Prognosis and Decision Support

After abnormalities have been detected, characterized, and quantified, the next step is to use imaging data to support clinical decisions. In this phase, AI is moving beyond image analysis alone, working as a decision-support tool that may help stratify patients better and guide management. In musculoskeletal oncology, these applications are developing rapidly. Quantitative imaging biomarkers from CT and MRI combined with ML models can predict treatment response and outcome.

The use of DL for outcome prediction in MSK oncology still remains limited. A few studies have investigated tasks such as recurrence prediction after curettage in giant cell tumor of bone using preoperative MRI. However, despite promising preliminary findings, the scarcity of large annotated datasets continues to limit the translation of most DL models into clinical practice [83]. In high-grade osteosarcoma, habitat imaging may identify intratumoral regions linked to response to neoadjuvant chemotherapy. Histogram-based features may reflect treatment-related changes in tumor structure. In soft-tissue sarcomas, simple MRI measures such as longest tumor diameter still remain clinically useful, but AI may add further prognostic information [84,85].

In degenerative and orthopedic imaging, DL and conventional ML approaches based on imaging data have been employed for prediction of structural osteoarthritis progression. In a subsequent multimodal study, CNNs were used to analyze raw DICOM radiographs and predict both current Kellgren-Lawrence grade and future osteoarthritis progression. CNN outputs were then fused with clinical data and Kellgren-Lawrence grade reaching an AUC of 0.81. Overall, this study shows that AI in osteoarthritis imaging is moving from automated grading toward multimodal risk prediction, where DL can extract information directly from raw images and classical ML can integrate imaging-derived outputs with clinical variables. However, external validation across different populations, acquisition settings, and non-standardized radiographic protocols remains essential before routine clinical adoption [86].

AI-based prognostic models are increasingly used for outcome prediction. In osteoarthritis, multimodal models that combine imaging biomarkers with clinical variables such as WOMAC scores may predict disease progression, symptom evolution, and the future need for joint replacement. In lumbar spine disease, AI may support risk stratification by combining imaging findings with demographic and clinical data. This is especially relevant in spine surgery, where multimodal models may help predict postoperative outcome, complication risk, and response to treatment [56].

Orthopedic implants are another important field for AI-based decision support. Postoperative implant evaluation follows a logical sequence that includes recognition of the anatomical region and radiographic view, detection of the implant, classification of implant type or model, measurement of implant position, and identification of complications [78,79,81]. More specifically, the process may begin with automated recognition of body part, laterality, and projection. It may then move to implant detection and classification of implant design or specific model. This is especially relevant in revision surgery planning, where implant-specific instruments may be needed, and correct implant identification may otherwise take time and remain uncertain. Some of these tasks are descriptive, but others have direct clinical value. AI can automatically measure implant-related parameters such as acetabular cup inclination and version, lumbar lordosis, and lower limb length [80,87,88]. This may improve speed and reproducibility. AI may also support a more structured postoperative assessment by linking technical measurements with clinically relevant findings. AI-based algorithms may also identify implant-related complications, particularly loosening, periprosthetic fractures, dislocation, infection, and component wear. To date, the accuracy of AI models in predicting these complications has remained moderate, partly because such events are relatively rare and training datasets are often unbalanced. Still, the combination of imaging and clinical data outperformed the use of imaging data alone. This suggests that implant-related AI may move from simple detection toward prediction and support for postoperative surveillance and revision planning [89].

Even in trauma, prognostic applications are starting to emerge. Most current models still focus on detection, but some studies are exploring imaging biomarkers related to ACL graft maturation or explainable models for vertebral compression fractures. Although these approaches are still in the early stage, they show that AI could contribute not only to diagnosis, but also to functional outcome prediction and treatment planning [90,91]. Compared with reconstruction and detection tasks, prognostic and decision-support applications remain less mature because they require longitudinal, multimodal, and clinically annotated datasets, as well as evidence that model outputs change patient management.

Prognosis and decision support are the domains in which AI is most closely linked to precision medicine. At the same time, these are among the least mature applications, because they require multimodal data integration, strong validation, and proof that the predictions truly improve clinical decisions. AI also has the potential to support reporting by helping radiologists create more structured reports and suggest appropriate follow-up recommendations [92]. This application is useful in daily practice, especially in high-volume settings. Its main value would not be to replace the radiologist’s judgment, but to improve standardization, reduce omission of relevant details, and support more consistent communication.

## 3. Challenges and Considerations

Despite its promise, the clinical use of AI in musculoskeletal imaging still faces important limitations. Pros and cons of AI applications in MSK imaging strongly depend on the specific technique, clinical task, input data, and level of integration into the radiological workflow, as summarized in Table 1. Therefore, AI should not be considered as a single homogeneous tool, but rather as a spectrum of methods with different strengths, computational requirements, and limitations. DL-based reconstruction and generative approaches are among the most clinically mature AI applications. Their main advantage is that they act in the early stage of the imaging chain, improving image quality, reducing acquisition time, supporting low-dose CT protocols, and increasing scanner efficiency. In MSK imaging, this may be particularly relevant because examinations are often time-consuming, patients may have pain-related motion, and repeated follow-up imaging is common. These tools can improve workflow without directly affecting the interpretative role of the radiologist. However, their integration into image generation also creates specific risks. DL reconstruction may alter image texture, suppress diagnostically relevant details, or produce images that appear visually convincing but do not perfectly reflect the underlying anatomy. Similarly, synthetic imaging and generative models may support radiation-free CT-like images generation, but they may also introduce hallucinated findings or reconstruction-related artifacts. For this reason, their apparent technical efficiency should be balanced against the need for careful validation and radiological oversight.

Detection and triage algorithms, particularly CNN-based models and object-detection architectures, offer different advantages. They are especially useful for high-volume and time-sensitive tasks, such as fracture detection, urgent abnormality flagging, and prioritization of examinations. Once trained and deployed, these models can provide rapid inference and may support less experienced readers, emergency physicians, or centers with limited access to subspecialized MSK radiologists. Their main clinical value is not only improved sensitivity, but also faster case prioritization and reduction in missed findings. However, their performance may decrease in subtle, atypical, postoperative cases and, on the other hand, false positives may increase radiologist workload. Therefore, detection algorithms are most useful when they function as second readers or triage tools, rather than autonomous diagnostic systems.

Radiomics-based models are particularly relevant for quantitative MSK imaging. They can automate the segmentation of muscle, fat, cartilage, bone and soft tissue tumors, allowing extraction of reproducible biomarkers that would be too time-consuming to measure manually in routine practice. Their advantage is the ability to generate pixel-level or voxel-level outputs, which can support objective quantification, longitudinal follow-up, and treatment monitoring. However, segmentation models usually require high-quality annotated datasets, and manual annotation remains time consuming, expert-dependent, and difficult to scale. In addition, their performance may be affected by acquisition protocols, image quality, anatomical variability, metal artifacts, and disease heterogeneity. Radiomics-based models are particularly useful in MSK oncology and rare diseases, but their translation into clinical practice is still limited by acquisition heterogeneity, feature redundancy, limited biological interpretability, and insufficient external validation.

Multimodal and ensemble approaches may offer additional value because they combine complementary sources of information. Ensemble models may improve prediction of multiple MSK conditions, including osteoarthritis progression, implant-related complications, or postoperative outcomes. Nevertheless, these systems are more complex to develop, validate, and deploy. They require larger and better-curated datasets and careful handling of missing data.

Interpretability remains one of the main limitations across nearly all AI techniques. Many DL systems still operate as “black boxes”, offering an output without providing the basis of the prediction. This is significant in MSK imaging, where findings may be subtle, multifactorial, and highly dependent on clinical context, prior surgery, or trauma. A false-negative AI output in fracture detection, for example, may lead to delayed diagnosis, while an unexplained false-positive output may increase unnecessary imaging, consultations, or patient anxiety. Explainable AI techniques, including Vision Transformers (ViT), saliency maps, heat maps, attention-based methods, and Shapley-based approaches, may partially improve transparency by showing which image regions or variables contributed to the model output. However, these methods should be interpreted cautiously, because they do not always provide a true causal explanation of the model. For this reason, explainability should be regarded as a support for radiological interpretation, not as a substitute for expert judgment [73].

Bias and poor generalizability represent another key limitation. As the performance of AI-based models is closely related to the quality and diversity of the training data, models developed on narrow populations or on technically homogeneous datasets may not perform as well when applied to different scanners, vendors, imaging protocols, or patient groups in everyday practice. In osteoarthritis research, for example, demographic reporting is often incomplete, and commonly used datasets may underrepresent some groups [93]. A major limit of the existing literature on AI in MSK imaging is the predominance of retrospective studies. A model that performs well in a curated dataset but requires excessive computational resources and lacks external validation may have limited practical value. AI medical devices have been mostly assessed retrospectively, with only a few prospective or randomized studies evaluating their real clinical impact. The lack of prospective, multicenter validation remains a critical gap, as AI algorithms may underperform significantly when tested on different cohorts and imaging scanners. Future studies should therefore report not only performance metrics, but also training and inference time, model size, hardware requirements, software environment, failure modes, and real-world clinical impact. This is essential to move AI from promising research tools to reliable support systems in daily MSK radiology practice.

The implementation of AI in MSK imaging also raises important ethical and medico-legal challenges. AI tools should not be evaluated only in terms of diagnostic accuracy, but also according to transparency, safety, fairness, reproducibility, clinical integration, and accountability.

In current clinical practice, AI systems should be considered decision-support tools rather than autonomous decision-makers. The radiologist remains responsible for the final interpretation and should be able to independently review the basis and plausibility of AI-generated recommendations. Blind reliance on an AI output may increase malpractice exposure, particularly if a human reader could reasonably have detected the abnormality missed by the algorithm. Conversely, radiologists may still need to justify why they accepted, rejected, or overrode an AI suggestion, especially if AI becomes progressively incorporated into departmental standards of care. This makes clear protocols for AI use essential, including procedures for managing human–AI discrepancy, documentation of AI outputs, user training, and periodic performance monitoring.

Liability may also extend beyond the individual radiologist. Hospitals, radiology departments, and private practices may be exposed through organizational or vicarious liability if an AI system is routinely used without adequate validation, governance, training, or workflow integration. In addition, AI developers and vendors may face product liability issues if software defects, insufficient validation, or unsafe deployment contribute to patient harm. This is complex because AI systems differ from traditional medical devices: they may require large external datasets, may be updated over time, and may not always provide interpretable outputs. These characteristics challenge conventional regulatory and liability frameworks and make post-market surveillance particularly important.

From a regulatory perspective, most AI tools have entered clinical use through approval pathways such as FDA clearance or CE marking, which often focus on safety and technical equivalence rather than direct evidence of improved patient outcomes. This creates a gap between regulatory approval and proven clinical value. Reporting standards also remain inconsistent, with limited transparency on safety, efficacy, software updates, failure modes, and post-market performance.

Ethical concerns also include privacy, data governance, equity, and fair access. AI development requires large imaging datasets. This increases the importance of anonymization, secure data storage, data-sharing agreements, and compliance with privacy regulations such as GDPR. At the same time, excessive restrictions on data sharing may limit external validation, creating a tension between privacy protection and model generalizability. Ethical AI development should therefore ensure that data are collected and used transparently, securely, and with attention to representativeness, avoiding models that reproduce existing inequalities or perform poorly in minority or underrepresented populations [94].

Another practical and ethical challenge is the annotation burden. High-quality labeled imaging data require expert MSK radiologist input, making dataset curation expensive, time-consuming, and difficult to scale. Furthermore, the environmental cost of training large AI models, including high computational energy consumption, is increasingly recognized but rarely reported.

These concerns can be framed within the Collingridge’s dilemma: in the early stages of a technology, its consequences are difficult to predict, whereas once the technology is widely adopted, it becomes much harder to regulate or modify. A proactive approach is therefore needed. Health Technology Assessment frameworks, including AI-adapted models such as MAS-AI, may help evaluate not only diagnostic performance, but also safety, clinical value, cost-effectiveness, ethical acceptability, organizational impact, and legal implications. Similarly, reporting tools such as CLAIM can improve transparency in AI studies by encouraging authors to describe data sources, model development, validation procedures, and potential sources of bias [95].

Overall, safe implementation of AI in MSK imaging requires more than regulatory approval or high retrospective accuracy. It requires continuous governance, prospective validation, explainability, bias monitoring, clear medico-legal responsibility, and evidence that AI improves real clinical outcomes. The radiologist should remain the final clinical decision-maker, with AI acting as an assistive tool that may improve efficiency, standardization, and detection performance, but only within a transparent and accountable framework.

Finally, the real clinical impact of AI is still not well demonstrated. Evidence for increased diagnostic accuracy in clinical practice is limited, workflow gains have not been assessed adequately, and strong cost-effectiveness analyses are still lacking.

These drawbacks underline the existing gap between models’ development and clinical translation, highlighting the need for rigorous, transparent, and outcome-driven research. The main challenge is not only to build accurate models, but also to make sure that they are reliable, fair, explainable, and clinically useful in the real world.

## 4. Conclusions

AI is changing MSK imaging at multiple levels, from image acquisition and reconstruction to detection, quantification, and decision support. Its main contribution is not only greater efficiency, but also a shift toward more standardized, quantitative, and clinically useful imaging assessment.

At present, the most mature applications appear to be those related to MRI reconstruction, fracture detection, and workflow support, because they fit more easily into routine clinical practice. Other areas, such as quantitative phenotyping, prognostic modeling, and multimodal decision support, are highly promising but still need broader external validation before they can be considered ready for wide clinical use.

For this reason, AI in MSK imaging should be seen as a tool that supports the radiologist, not as a substitute for radiological expertise. Its future value will depend not only on technical progress, but also on careful validation, transparent governance, and close collaboration between radiologists, clinicians, and AI researchers. If developed and used responsibly, AI may help make MSK imaging faster, more reproducible, and more informative for patient care.

## Figures and Tables

**Table 1:** Overview of AI applications in musculoskeletal imaging.

Application Domain	Typical Tasks	Main Advantages	Key Limitations
Acquisition and reconstruction	MRI acceleration, denoising, artifact reduction, low-dose CT reconstruction	Reduced scan time, improved image quality, reduced radiation dose	Limited transparency of reconstruction processes
Detection and triage	Fracture detection, soft tissue injuries identification, bone lesion detection	Faster reading, improved sensitivity (especially for subtle findings), prioritization of urgent cases	Variable performance across datasets, limited interpretability (“black box”)
Characterization and quantification	Body composition analysis (muscle and fat segmentation), cartilage analysis, bone density estimation, radiomics feature analysis	Objective and reproducible measurements, automated analysis, potential for quantitative biomarkers	Reproducibility issues, lack of standardization, uncertain clinical integration
Prognosis and decision support	Outcome prediction, risk stratification, surgical planning, implant assessment	Integration of imaging and clinical data, potential support for personalized medicine	Limited prospective validation, unclear impact on patient management

## Data Availability

No data were analyzed.

## Abbreviations

The following abbreviations are used in this manuscript:

AI	Artificial Intelligence
MSK	Musculoskeletal
ML	Machine Learning
DL	Deep Learning
CNN	Convolutional Neural Network
CT	Computed tomography
MRI	Magnetic resonance imaging
AUC	Area under the curve
